# Flavonoids as Insecticides in Crop Protection—A Review of Current Research and Future Prospects

**DOI:** 10.3390/plants13060776

**Published:** 2024-03-08

**Authors:** Verónica Pereira, Onofre Figueira, Paula C. Castilho

**Affiliations:** CQM—Centro de Química da Madeira, Universidade da Madeira, Campus da Penteada, 9200-105 Funchal, Portugal

**Keywords:** biopesticides, flavonoids, insect control, mode of action

## Abstract

Pesticide overuse in agricultural systems has resulted in the development of pest resistance, the impoverishment of soil microbiota, water pollution, and several human health issues. Nonetheless, farmers still depend heavily on these agrochemicals for economically viable production, given the high frequency at which crops are affected by pests. Phytopathogenic insects are considered the most destructive pests on crops. Botanical pesticides have gained attention as potential biopesticides and complements to traditional pesticides, owing to their biodegradability and low toxicity. Plant-based extracts are abundant in a wide variety of bioactive compounds, such as flavonoids, a class of polyphenols that have been extensively studied for this purpose because of their involvement in plant defense responses. The present review offers a comprehensive review of current research on the potential of flavonoids as insecticides for crop protection, addressing the modes and possible mechanisms of action underlying their bioactivity. The structure–activity relationship is also discussed. It also addresses challenges associated with their application in pest and disease management and suggests alternatives to overcome these issues.

## 1. Introduction

Insects are considered the main pests responsible for crop losses worldwide because of their dual capacity to feed on plant tissues by chewing or sap-sucking and act as vectors of plant viruses [1,2]. The Green Revolution of the mid-1900s changed global agriculture by introducing innovative approaches to enhance crop productivity and reduce pest infection [3]. One of these measures corresponded to the introduction of pesticides as crop protectants. Although farmers depend heavily on pesticides to have an economically viable production, direct or indirect human exposure to pesticides has strongly promoted health diseases and disorders. These health issues include the development of neurodegenerative diseases (for example, Alzheimer’s and Parkinson’s) [4,5], several types of cancer (such as breast, colorectal, lung, and prostate cancers) [6,7,8,9,10,11], and alterations to the reproductive system (from genital malfunctions to decreased fertility) [12,13,14]. In nature, pesticide overuse has raised concerns regarding the sustainability of agricultural systems. Pesticides affect soil quality because of their ability to degrade organic matter and damage microbial biodiversity by disrupting the interactions between microorganisms and plant roots or by affecting the nitrogen cycle [15,16]. Water-soluble pesticides reach groundwater by leaching downward into soil layers. Those insoluble compounds bind to soil particles and are susceptible to water runoff and soil erosion, thereby contaminating lakes and rivers [17]. In addition, pesticide efficiency has reduced significantly owing to the development of multiple pest resistance mechanisms, which result from genomic and transcriptomic changes [18,19]. Beyond the evident impact on crop productivity, the economic impact also includes the costs associated with the application of ineffective insecticides. Despite the well-known adverse effects associated with the use of traditional pesticides, their application is not fully prohibited, as they still play an important role in controlling pests associated with the transmission of diseases. For example, dichlorodiphenyltrichloroethane (DTT) is a broad-spectrum and highly active insecticide that accumulates in the environment because of its high stability, leading to toxicity in animals and humans. Nonetheless, it cannot be ignored that DTT helped in controlling and eradicating malaria in Europe and the United States, given its high insecticide activity against the malaria mosquito *Anopheles* (Meigen, 1818) [20]. Facing this fact, researchers and farmers have worked together to adopt integrated pest management strategies that do not compromise human and environmental health, economic profitability, and social equity. These strategies focus on minimizing the use and concentration of applied pesticides while maximizing production and maintaining sustainable agriculture by encouraging the utilization of natural products and biological organisms as pest control agents, known as biopesticides [21]. The interest in sustainable and safer pest control was also potentiated by initiatives such as the European Union (EU) Green Deal, which, among other goals, aims to reduce the use of chemical pesticides by 50% by 2030 [22].

During their evolution, plants have developed the capacity to produce a wide diversity of secondary metabolites, which they utilize in their responses and adaptation mechanisms to biotic and abiotic factors. In light of this situation, these compounds have attracted attention as potential biopesticides. Known as botanical pesticides, they are commonly used as essential oils or plant-based extracts, being preferred over conventional pesticides for their simple preparation and affordability. For instance, aqueous and ethanolic extracts prepared by maceration are the most commonly studied as bioinsecticides [23]. As insecticides, they act especially on the nervous system of insects by affecting neuronal channels (such as sodium and γ-aminobutyric acid-gated chloride channels), receptors (nicotinic acetylcholine, octopamine, and tyramine receptors), or even enzymes (like acetylcholinesterase) [15]. They can also inhibit respiratory enzymes, affect the digestive system, and bind to specific muscular receptors. These activities are based on the wide variety of bioactive molecules that they can contain. In plant-based extracts, alkaloids, phenolic acids, flavonoids, saponins, sterols, tannins, and other compounds can be identified. 

**Figure 1:**
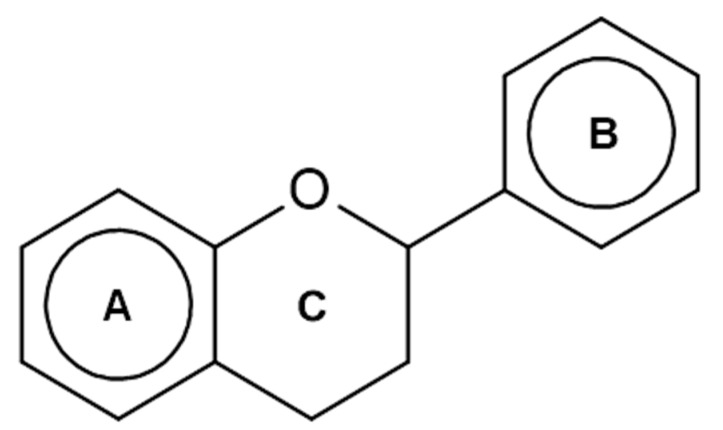
Flavonoid chemical structure (phenyl rings are represented by the letters A and B, and the heterocyclic pyran ring by letter C).

Flavonoids are the most abundant non-nitrogenous phytochemicals found in vascular plants, comprising 10,000 known secondary metabolites in the Plant Kingdom [24,25]. They are synthesized via a mist pathway and accumulate in the cell vacuoles of plant-specific organs such as leaves and fruits, where they have a wide diversity of physiological functions. For example, flavonoids regulate plant development and pigmentation; protect plants against ultraviolet radiation, insects, and pathogens; function as signaling molecules during nodulation; and regulate auxin transport and male fertility [26,27,28]. These polyphenols comprise the largest group of non-enzymatic antioxidants produced by plants under stress conditions, regardless of whether they are induced by biotic or abiotic factors. Their abundance in these situations is mainly because these molecules are reducing agents [26]. Structurally, the flavonoid core is usually referred to as C6-C3-C6 because it consists of two phenyl rings connected through a heterocyclic pyran ring (Figure 1). Each subclass differs from the others in terms of the level of oxidation, unsaturation, and pattern of substitution of the C ring, whereas those belonging to the same subclass differ in the substitution of rings A and B. They can be found in plants in their free form, designated as aglycone, glycosylated, or methylated [24].

Flavonoids have been revised as promising biopesticides but those revisions lacked in reporting the modes and mechanisms of action underlying their insecticide activity [29]. The present literature review provides an overview of the current knowledge on flavonoids as insecticides in crop protection. For this purpose, a brief presentation of the general synthesis pathway of flavonoids in plants and originating subclasses is presented, along with their distribution throughout the plant kingdom. A summary of the main research findings on various flavonoid subclasses as insecticides is presented, highlighting their form of application, trends in target organisms tested, and the main crops affected by them. Each section discusses the modes of action discovered and the functional groups in the chemical structure reported to be responsible for their bioactivity. This review addresses the main challenges associated with their application in pest and disease management and provides future perspectives in this line of research.

## 2. Flavonoid Biosynthesis Pathway, Subclasses, and Distribution

**Figure 2:**
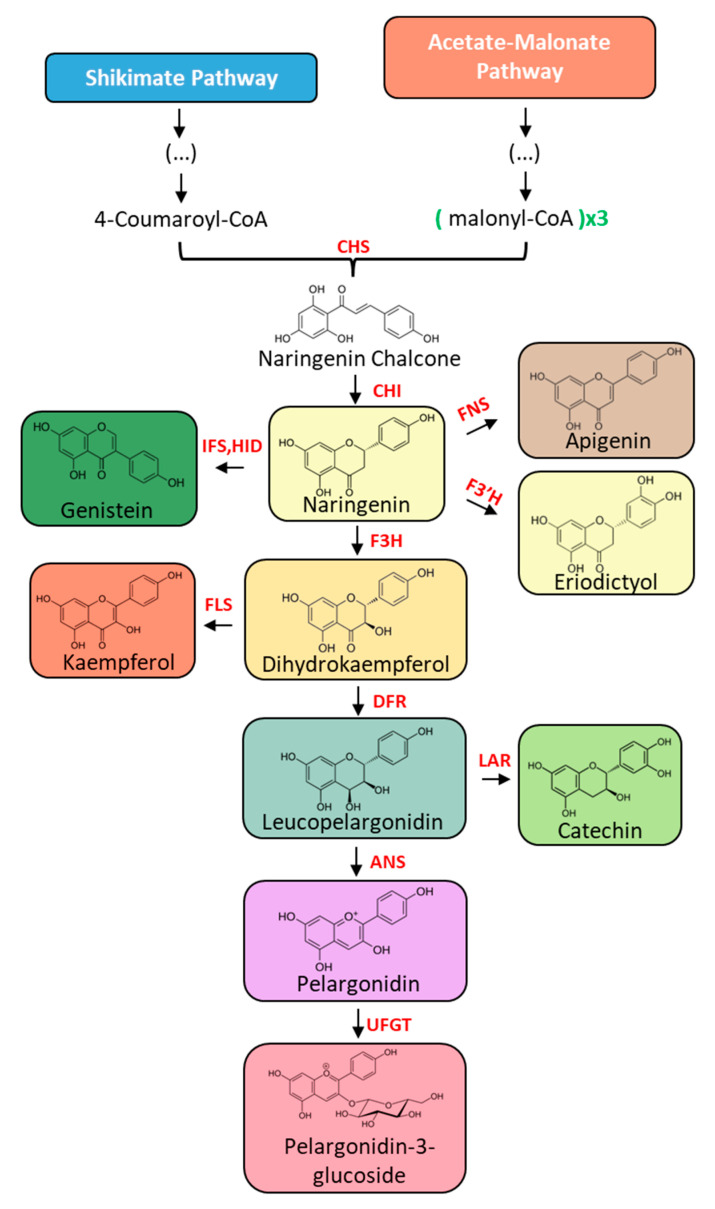
General flavonoid biosynthesis pathway. Each block color represents a different flavonoid subclass. Legend: CHS, chalcone synthase; CHI, chalcone isomerase; FNS, flavone synthase; F3′H, flavanone 3′-hydroxylase; IFS, isoflavone synthase; HID, 2-hydroxyisoflavanone dehydratase; F3H, flavanone 3-hydroxylase; FLS, flavonol synthase; DFR, dihydroflavonol 4-reductase; LAR, leucoanthocyanidin reductase; ANS, anthocyanidin synthase; UFGT, UDP flavonoid glucosyl transferase.

Figure 2 provides a generalized view of the flavonoid biosynthesis pathway in plants, in which each color represents a different flavonoid subclass. The biosynthesis pathway and diversity of subclasses of flavonoids existing in plants are highly dependent on their genetic code and consequent enzyme expression. Yet, the initial step of this pathway remains the same across all species, which corresponds to the condensation of substrate 4-Coumaroyl-CoA, synthesized via the shikimate pathway, with three molecules of malonyl-CoA, derived from acetate–malonate pathway. This reaction is mediated by the enzyme chalcone synthase (CHS), originating from naringenin chalcone, the first intermediate of this pathway [30]. Following this, this intermediate suffers an intramolecular cyclization mediated by chalcone isomerase (CHI), resulting in the flavanone naringenin (represented in light yellow) [27,31]. This flavanone is the first compound of the pathway to exhibit the 3-ring flavonoid skeleton (Figure 1) from which structures diverge through the action of different enzymes. For instance, it can generate other flavanones (such as eriodyctiol, represented in light yellow), flavones (like apigenin, represented in brown), isoflavones (for example, genistein, represented at dark green), and dihydroflavonoids (such as dihydrokaempferol, represented in orange) [27,32,33,34,35]. From this last subclass, flavonols (represented in light red, such as kaempferol) can be obtained but also leucoanthocyanins [27,36,37]. Flavonoids belonging to this last subclass can act as precursors for the synthesis of flavan-3-ol (represented in light green, such as catechin) and anthocyanidins (represented in purple, like pelargonidin), which can consequently give origin to anthocyanins (represented in pink, like pelargonidin-3-glucoside) [27,38,39].

Flavonoids are well distributed throughout almost the entire Plant Kingdom and have been identified in nonvascular plants, such as liverworts and mosses, and vascular plants, such as lycophytes, ferns, gymnosperms, and angiosperms [28]. Flavones and flavanones are present in vascular and nonvascular plants, while other subclasses are found in specific types, demonstrating the diversification and evolution of plants. For example, to date, isoflavones have only been found in gymnosperms and angiosperms. Although it is generally accepted that the flavonoid pathway is restricted to terrestrial plants, flavonoids have already been identified in aquatic plants, such as *Nelumbo nucifera*, and a biosynthesis pathway proposed [40].

## 3. Flavonoids as Botanical Insecticides

Table 1 summarizes several studies that have shed light on the insecticidal potential of different flavonoids against various global pests. Flavones and flavanols were found to be the most studied subclasses, given their higher abundance and availability in plants. In most studies, flavonoids were incorporated at a certain concentration in an insect diet, either in artificial ones [41,42,43], such as gels [44,45], or by application in leaf surfaces via dipping [46,47,48]. Less commonly, flavonoids were injected directly into the larvae using a syringe [49,50]. All these studies focused on pests relevant to food crops, with the exception of *Macrosiphoniella sanborni*, which is a flower crop aphid [51]. Based on our research, the target model organisms range from species of caterpillars (such as *Spodoptera litura* (Fabricius, 1775), *Spodoptera frugiperda* (J.E.Smith, 1797), and *Helicoverpa zea* (Boddie, 1850)), flies (e.g., *Bemisia tabaci* (Gennadius, 1889), *Zeugodacus cucurbitae* (Coquillett, 1899), and *Bactrocera cucurbitae* (Coquillett, 1899)), beetles (like *Epilachna paenulata* (Chevrolat, 1836) and *Tribolium castaneum* (Herbst, 1797)), moths (e.g., *Plutella xylostella* (Linnaeus, 1767), *Mythimna separata* (Walker, 1865), and *Ostrinia furnacalis* (Guenée, 1854)), and aphids (such as *Aphis gossypii* (Glover, 1877), *Acryrthosiphon pisum* (Harris, 1776), and *Macrosiphoniella sanborni* (Gillette, 1908)). The main results obtained and the proposed mode of action depended heavily on the structure and concentration of the flavonoid, the model insect, and methodology applied.

### 3.1. Feeding Disruptors

Flavonoids are commonly reported as feeding deterrents in all insect types (Table 1). This mode of action allows an insect to die through starvation if the insect remains near the treated leaves, and is of high importance, especially to control insect larvae, the stages of which are more destructive to crops. Ohmura et al. [64] studied the antifeeding activity of flavonoids against the termite *Coptotermes formosanus* (Shiraki, 1909) and suggested that the structure–activity relationship underlying a higher activity resided on the hydroxylation of positions C5 and C7 in the A ring and C3′ and C4′ in the B ring, and in the presence of a carbonyl group at position C4 in ring C. Later, Morimoto et al. [65] predicted that the feeding deterrence of flavonoids against *S. litura* depended on the pattern of substitution of the A ring, with the antifeeding activity being higher when introducing a substituent at position C6 or C7. Moreover, the presence of positive and negative charges at positions C3 and C5, respectively, could be responsible for the antifeeding activity. Although flavonoids seem to have a great impact on insect feeding behavior, especially in caterpillars, only Goławska et al. [44] and Stec et al. [56] reported some positive effects of flavonoids on the probing behavior of aphids (Table 1). Moreover, some studies show that flavonoid antifeeding activity acts in a concentration-dependent manner and that at low concentrations, they can act as a phagostimulant, which was the case for pinocembrin and quercetin (Table 1) [54,55]. These observations are of equal importance, as these compounds can be used as bait at phagostimulant concentrations in less economically valuable crops, thereby protecting the most valuable crops. These compounds can eventually be combined with traditional insecticides to reduce or control pest populations.

Given the role of flavonoids as feeding deterrents, it is reasonable to assume that the mechanism of action underlying this effect may rely on the inhibition of key digestive enzymes in insect midguts, which are amylases, glycosidases, lipases, and proteases. War et al. [66] evaluated the inhibitory activity of three flavonoids (catechin, quercetin, and trihydroxyflavone) on serine protease and trypsin isolated from *H. armigera* and observed that trihydroxyflavone significantly reduced the total serine protease and trypsin enzymatic activities. Another study assessed the inhibitory activity of six flavonoids (catechin, kaempferol, myricetin, naringenin, quercetin, and rutin) on both of these enzymes but isolated from *S. litura* [67]. For all tested flavonoids, larvae fed at 1000 ppm revealed a lower serine protease activity than those fed on other treatments or the untreated ones. Naringenin- and catechin-fed larvae did not exhibit significantly lower trypsin activity at 1000 ppm. Mikani [68] demonstrated that quercetin decreased the activity of lipase (from 98.4 mU to 62.5 and 44.8 mU for 500 and 1000 ppm, respectively), protease (from 80 mU to 40.7 and 40.2 mU for 500 and 1000 ppm, respectively), and α-amylase (from 150 mU to 72 and 55.2 mU for 500 and 1000 ppm, respectively) in the midgut of *P. xylostella*. Later, Maazoun et al. [69] studied the inhibitory effect of an *Agave americana* leaf extract abundant in flavonoid glycosides (kaempferol, quercetin, and isorhamnetin derivates) on α-amylase and protease isolated from rice weevils. The authors verified that this flavonoid-rich extract significantly inhibited these enzymes in a concentration-dependent manner, with IC_50_ values of 146.06 ± 1.74 and 86.18 ± 1.08 μg/mL for α-amylase and protease, respectively [69].

### 3.2. Detoxification System Disruptors

Detoxifying enzymes are responsible for transforming xenobiotics or toxic compounds into less or nontoxic compounds that are prone to excretion, protecting the insect from damage. Larvae fed on flavonoids suggested the induction of oxidative stress due to an increase or decrease in detoxifying enzyme activities. For instance, Punia and Chauhan [63] observed that *S. litura* larvae treated with daidzein presented significantly higher enzymatic activities of superoxide dismutase, catalase, ascorbate peroxidase, and glutathione S-transferase, indicating oxidative stress and the need to metabolize daidzein. The authors also verified an increase in oxidative stress markers, namely hydrogen peroxide, lipid peroxide, and protein carbonyl. War et al. [66] reported a higher activity of glutathione S-transferase in *H. armigera* when fed diets containing trihydroxyflavone and catechin at 1000 ppm than at 500 ppm and the relative control. However, esterase activity was significantly lower in the assays performed with 1000 ppm of catechin than at 500 ppm. These biochemical alterations could interfere with food uptake by larvae because the energy used to digest food could be transferred to detoxify these flavonoids [63]. On the other hand, the inhibition of these enzymes could result in greater toxicity to the organism. Wang et al. [70] showed that flavanonol taxifolin was able to inhibit *Leptinotarsa decemlineata* (Say, 1824) esterase in vitro (IC_50_ of 2.6 ± 0.3 mg/L). In vivo studies revealed that taxifolin significantly reduced esterase activity but slightly decreased glutathione-S-transferase activity. Another study demonstrated that chrysin and galangin reduced the carboxylesterase activity of *S. litura*, an enzyme associated with insecticide resistance, while increasing the activity of glutathione-S-transferase (Table 1) [50]. The combination of all these possible mechanisms of action may also justify the recurring mention of a significantly higher mortality rate when insects are treated with flavonoids. 

### 3.3. Growth, Development, and Reproduction Disruptors

In addition to their antifeeding and phagostimulatory effects, flavonoids have been reported to delay development and growth, induce malformations, and affect the reproductive cycle of adult insects by increasing the pre-reproductive period and decreasing fecundity, oviposition, and egg hatching (Table 1) [42,44,48,49,55,59,60]. For several years, acetylcholinesterase (AChE) has been a target enzyme in pesticide research because of its well-known role in cholinergic neurotransmission, and its inhibition could be responsible for this physiological effect. Li et al. [71] suggested that the growth and development of *S. litura* larvae were affected when fed with several flavonoids owing to their AChE inhibitory capacity. The authors speculated that the higher inhibitory activity could be due to additional phenolic hydroxyl groups in glucose at position C7 and the degree of hydroxylation on both rings A and B. For these reasons, quercetagetin-7-O-(6-O-caffeoyl-β-D-glucopyranoside) had the highest inhibitory activity against AChE from *S. litura*, with an IC_50_ of 12.58 µg/mL. In addition, Kumar et al. [72] showed that this enzyme could be involved in the embryonic development of *H. armigera* larvae, given that those fed with siRNA reached normal pupal stages but were malformed, and the respective adults presented a significantly lower egg hatching rate. Therefore, AChE inhibition could also be a mechanism of action for flavonoids as insecticides. Nevertheless, some flavonoids also seem to stimulate oviposition [73,74,75] and, therefore, could be used in insect traps. Angiotensin-converting enzyme (ACE) and juvenile hormone (JH) acid methyltransferase (JHAMT) are reported to be important enzymes in the insect reproductive system but no reports were found of the inhibitory activity of flavonoids on these insect enzymes [76,77]. 

Molting is a vital process for insects, where they periodically renew their exoskeletons through the shedding and formation of new chitinous cuticles to allow their growth and development. This cuticle structure protects insects from microbial infections, dehydration, and physical injuries. Loss or inhibition of chitin synthesis and/or degradation enzymes results in exoskeleton defects and lethality, independent of the insect stage [78,79]. Therefore, insecticide design and research aimed at inhibiting chitinolytic enzymes have received increasing attention. Only Li et al. [41] have studied the capacity of flavonoids, in particular three flavones (baicalein, chrysin, and wogonin) and four flavonols (galangin, quercetin, myricetin, and kaempferol), to inhibit chitinolytic enzymes from *O. furnacalis* (*Of*ChtI, *Of*ChII, *Of*Chi-h, and *Of*Hex1). Their inhibitory kinetics data revealed that only baicalein, quercetin, and myricetin inhibited all tested enzymes. The authors attempted to unravel which differences in the molecular structure justified why other flavonoids outperformed baicalein for specific enzymes. Through molecular modeling, they verified that the presence of C3′ and C5′ hydroxyl groups increased the established hydrogen bonds with residues of the active-site pocket of OfHex1, and that their inhibitory activity was positively affected by the number of hydroxyl groups on the B ring (myricetin > quercetin > kaempferol > baicalein). In addition, higher enzymatic inhibition could be related to C8-methoxyl substitution (wogonin > baicalein) and stronger π−π stacking interactions (myricetin > baicalein).

### 3.4. Nervous System Disruptors

As shown in the previous section, flavonoids can act on the nervous system of insects by affecting crucial enzymes such as AChE, but they can also affect neuronal channels, despite being less extensively studied. For instance, Ren et al. [80] showed that the mechanism underlying the insecticide activity of three biflavones was related to their capacity to inhibit different voltage-gated potassium channels from the ventral nerve cord of *H. zea* and *Heliothis virescens* (Fabricius, 1777) via gating modification.

## 4. Challenges and Prospects

Flavonoids show great potential to be employed as crop protectants against insects as shown and discussed in previous sections. Bearing this in mind, their commercialization and field application are targets of interest. Interestingly, a patent has already been registered for the isoflavone formononetin in combination with traditional insecticides (U.S. Patent No. 8334268) [81]. However, flavonoids face some limitations that can restrain their field application. One of the most important aspects to take into consideration is their overall toxicity for non-target organisms. Most studies report flavonoid efficiency at a given concentration against a target organism but lack in studying their effect on plant and soil beneficial ones. These organisms can include pollinating insects, organic matter decomposers, nitrogen-fixing bacteria, mycorrhizal fungus, and others. For example, Selin-Rali et al. [61] concluded that quercetin presented lower toxicity on the earthworm *Eisenia fetida* (Savigny, 1826) than the common synthetic insecticides chlorpyrifos and cypermethrin. Furthermore, studying and establishing a possible mechanism of action for these phenolic compounds against an insect are crucial because the target enzyme may be common to other insects or even mammals. This is the case of currently used insecticides that target the catalytic serine residue of acetylcholinesterase, which is their key residue that regulates acetylcholine levels in both vertebrates and invertebrates. In light of this situation, pesticide research focusing on cysteine-targeting acetylcholinesterase, unique to insects, has been a target of interest to minimize unintended toxicity [82].

Although advocated as biodegradable molecules given their natural origin, another important aspect to bear in mind is their biodegradability rate and fate on soil. Similar to currently used pesticides, their field application can be affected by atmospheric conditions, such as rain and wind, which can result in their accumulation in soil and possible negative effects in soil microbe populations. Ozan et al. [83] observed that microorganisms present in nonsterile soil were able to metabolize the isoflavones formononetin and biochanin A, with both being recovered in about 20 and 60% of the initial concentration, respectively, after 15 days of incubation. Later, Shaw and Hooker [84] concluded that naringenin and formononetin were rapidly biodegraded in soil and did not inhibit soil microbial dehydrogenase activities. Nonetheless, more representative studies in terms of soil biodiversity should be conducted.

Biopesticides, in general, suffer from premature degradation owing to their rapid biodegradability due to their contact with atmospheric conditions, resulting in a lack of efficiency and stability. Their rapid biodegradability and low efficiency when compared to traditional pesticides limit their application, and, therefore, biopesticides have been suggested as complements to conventional pesticides to reduce their concentration applied. To overcome these issues, researchers have focused on their encapsulation in biodegradable polymers. Enclosing a pesticide within a material allows for a controlled release over time and targeted delivery, which reduces pesticide loss and provides less exposure of non-target organisms to pesticides, making them safer for the environment and human health. For instance, the nanoencapsulation of insecticides could be beneficial for increasing efficiency, given that they are more easily absorbed into the lipid layers of insects, disrupting the water protection barrier and causing insect death via desiccation [85]. In the specific case of flavonoids, their entrapment in hydrophilic polymers could also enhance their water solubility, decreasing the ecological impact associated with dissolution in organic solvents. However, the nanoencapsulation of flavonoids for insecticide application has only been performed in the form of an extract in combination with other phenolic compounds. Therefore, flavonoid encapsulation for this application could constitute an important line of research.

## 5. Conclusions

Flavonoids show great potential as insect-controlling agents because of their ability to interfere with digestion, growth, development, and reproduction, whether by inhibiting or stimulating them. However, further research is required to determine whether these molecules at a given concentration can be detrimental to nontoxic organisms as well as their impact on the soil and biodegradability rate. If these concerns are addressed, patenting and the consequent commercialization will become easier and safer.

## Figures and Tables

**Table 1:** Broad description of flavonoid subclasses and respective examples researched as insecticides for specific target organisms and the main results. Legend: LD_50_, 50% lethal concentration; AFC_50_, 50% antifeedant concentration.

Flavonoid	Target Organism	Main Affected Crops	Main Results	Reference
Flavan-3-ol				
Catechin	*S. litura*	Tobacco, Cotton	LD_50_ of 8.37 µg/2nd-instar larvae after 24 h. It decreased the activities of acetylcholinesterase, carboxylesterases, and glutathione S-transferase in the larvae.	[52]
Epigallocatechin Gallate	*A. gossypii*	Melon, Cucurbits, Cotton	For Cucurbit- and Cotton-specialized aphids, it inhibited development, survival, and fecundity at 10 mg/L.	[48]
Flavanone				
Hesperidin	*B. tabaci*	Sweet potato, Cabbages, Avocado	[Mg(hesp)_2_(phen)]OAc (0.14 µmol/L) killed 80% of adults whitefly after 72 h.	[53]
	*S. frugiperda*	Maize, Soy, Cotton	[Mg(hesp)_2_(phen)]OAc (0.14 µmol/L) killed all 2nd-instar larvae after 72 h.	
			[Cu(phen)(hesperidin)] increased larval mortality by 96.66% when compared to control.	[46]
Naringenin	*A. pisum*	Pea and other leguminous	Increasing concentrations increased the development time, the pre-reproductive period, and mortality, and decreased fecundity. At 1000 µg/cm^3^, it blocked ingestion and deterred aphid probing.	[44]
Pinocembrin	*E. paenulata*	Cucurbits	Pinocembrin (5 and 50 µg/cm^2^) stopped larval feeding after 9 days. Mechanism of action through chronic intoxication for food-deprived larval.	[54]
	*S. frugiperda*	Maize, Soy, Cotton	High concentrations (5 and 50 µg/cm^2^) were rejected by 3rd-instar larvae. At low concentrations (0.1, 0.01, and 1 µg/cm^2^), it acted as a phagostimulant, while at high concentrations (10, 50, and 100 µg/cm^2^), it acted as a deterrent.	[55]
Flavone				
Apigenin	*A. pisum*	Pea and other leguminous	At 0.1%, it reduced probes in general (2.5 times lower) but enhanced the duration of probes in non-phloem tissues (2.3 times longer than on control).	[56]
	*S. litura*	Tobacco, Cotton	At 100 µg/mL, it had a mortality rate of 50% after 48 h on 2nd-instar larvae. At 500 µg/mL, it reduced antifeedant activities by approximately 1.2-fold when compared to control.	[47]
Chrysin	*S. litura*	Tobacco, Cotton	LD_50_ of 2.752 µg/2nd-instar larvae. Reduced carboxylesterase activity (approximately 1.2-fold reduction) and induced glutathione-S-transferase activity (1.2-fold induction)	[50]
	*Z. cucurbitae*	Melon	Chrysin (3125 ppm) was more effective in reducing percent pupation for the 1st-instar larvae (53.02%) than in the 2nd and 3rd instars (46.15 and 11.49%, respectively). Oviposition was reduced under choice and no-choice conditions.	[42]
Cynaroside	*M. sanborni*	*Chrysanthemum*	LC_50_ of 7.4207 mg/mL after 4 h for 3rd-instar larvae.	[51]
	*M. separata*	Maize, Rice, Wheat	At 1 mg/mL, it had a mortality rate of 36% on 3rd-instar larvae.	
	*P. xylostella*	Cruciferous vegetables	AFC_50_ of 0.0109 mg/mL and LC_50_ of 0.0703 mg/mL after 48 h for 3rd- and 4th-instar larvae, respectively.	
Luteolin	*A. pisum*	Pea and other leguminous	Passive ingestion and salivation were completely blocked at 100 µg/cm^3^.	[45]
	*M. sanborni*	*Chrysanthemum*	LC_50_ of 24.0429 mg/mL after 4 h for 3rd-instar larvae.	[51]
	*P. xylostella*	Cruciferous vegetables	AFC_50_ of 0.1462 mg/mL and LC_50_ of 0.0047 mg/mL after 48 h for 3rd- and 4th-instar larvae, respectively.	
	*S. litura*	Tobacco, Cotton	At 100 µg/mL, it had a mortality rate of 18% after 48 h on 2nd-instar larvae. At 500 µg/mL, it reduced antifeedant activities by approximately 1.3-fold when compared to control.	[47]
Wogonin	*M. separata*	Maize, Rice, Wheat	At 10 mM, it had a mortality rate of 88% after 6 days.	[41]
	*O. furnacalis*	Maize	Mortality rate around 50% and reduced larval weight after 6 days.	
	*S. frugiperda*	Maize, Soy, Cotton	Significant changes in mortality rate were not detected. Larval weight decreased by 38% compared to those in the control group.	
Flavonol				
Galangin	*S. litura*	Tobacco, Cotton	All concentrations (5, 25, 125, and 625 ppm) reduced the capacity of 2nd-instar larvae to gain weight.	[57]
			LD_50_ of 4.718 µg/2nd-instar larvae. Reduced carboxylesterase activities (approximately 1.4-fold reduction) and induced glutathione-S-transferase (1.5-fold induction).	[50]
Kaempferol	*A. pisum*	Pea and other leguminous	At 0.1%, it prolonged the non-probing phase (3.5 times longer than the control).	[51]
	*M. sanborni*	*Chrysanthemum*	LC_50_ of 6.2688 mg/mL after 4 h for 3rd-instar larvae.	
	*M. separata*	Maize, Rice, Wheat	At 1 mg/mL, it had a mortality rate of 82% on 3rd-instar larvae.	
	*P. xylostella*	Cruciferous vegetables	AFC_50_ of 0.0122 mg/mL and LC_50_ of 1.0586 mg/mL after 48 h for 3rd- and 4th-instar larvae, respectively.	
Quercetin	*A. pisum*	Pea and other leguminous	Increasing concentrations of quercetin increased the development time, the pre-reproductive period, and mortality, and decreased fecundity. Quercetin deterred aphid probing and feeding.	[44]
			On *Pisum sativum*, quercetin (0.1 and 0.5%) did not affect the aphid probing behavior when compared to control.	[58]
	*B. cucurbitae*	Melon	At 3125 ppm, it reduced egg hatching (to 86.20% of the control), larval and pupal weight of the 2nd instar (5.4 and 8.8, respectively), percentage pupation and emergence of all instars (47 and 25%, respectively), and food assimilation.	[59]
	*E. paenulata*	Cucurbits	Acted as a phagostimulant.	[54]
	*M. sanborni*	*Chrysanthemum*	LC_50_ of 18.4179 mg/mL after 4 h for 3rd-instar larvae.	[51]
	*M.separata*	Maize, Rice, Wheat	At 1 mg/mL, it presented a mortality rate of 86% on 3rd-instar larvae.	
	*Myzus persicae* (Sulzer, 1776)	Peach, Potato	On *Brassica rapa* subsp. *pekinensis*, quercetin (0.1 and 0.5%) did not affect the aphid probing behavior when compared to control.	[58]
	*P. xylostella*	Cruciferous vegetables	AFC_50_ of 0.0242 mg/mL and LC_50_ of 0.0696 mg/mL after 48 h for 3rd- and 4th-instar larvae, respectively.	[51]
	*Rhopalosiphum padi* (Linnaeus, 1758)	Wheat, Barley, Oat, Rye	On *Avena sativa*, quercetin (0.1 and 0.5%) did not affect the aphid activities’ probing behavior when compared to control.	[58]
	*S. frugiperda*	Maize, Soy, Cotton	Higher deterrent effects with increasing concentrations when compared to control. At low concentrations (0.1, 0.01, and 1 µg/cm^2^), it acted as a phagostimulant.	[55]
	*S. litura*	Tobacco, Cotton	At 1 µg/mL, it provided the lowest larval survival percentage after 24 days. All the tested concentrations had the same extended larval duration (around 40 days).	[60]
			At 50 ppm, it had the highest mortality rate for all tested larvae instars. It negatively affected larvae growth and pupae weight. Quercetin’ s effect on earthworm was non-significant when compared with monotrophos and cypermethrin.	[61]
Rutin	*A. pisum*	Pea and other leguminous	On *Pisum sativum*, it only significantly increased the duration of time needed to achieve the first sustained sap ingestion period (1.8 and 2.5 times longer for 0.1 and 0.5%, respectively).	[58]
	*Helicoverpa armigera* (Hübner, 1808)	Pigeon pea	At 1 µg/mL, it had the lowest larval survival and weight percentages after 21 days, and the highest larval duration and extension (32 and 51 days, respectively) due to cessation of feeding.	[60]
	*M. persicae*	Peach, Potato	On *Brassica rapa* subsp. *Pekinensis*, the first phloem phase was 3.3 times longer, the number of probes was 1.5 times higher, and the duration of probes was 3.0 times lower for 0.5% rutin-treated plants.	[58]
	*R. padi*	Wheat, Barley, Oat, Rye	On *Avena sativa*, in 0.5% rutin-treated plants, more aphids reached sieve elements and sooner than the control and 0.1% concentration.	
	*S. litura*	Tobacco, Cotton	All concentrations extended larval duration to between 27 and 52 days. Significant effect on larval development, pupal mortality, and malformed adults.	[60]
Isoflavone				
Daidzein	*A. pisum*	Pea and other leguminous	At 0.1%, it delayed the ability of aphids to reach phloem vessels (3 times more time than the control) and limited sap ingestion (49% at the end of the experiment).	[56]
	*S. litura*	Tobacco, Cotton	No antifeedant activity against 4th-instar larvae but inhibited its growth after 3 days.	[62]
			At 625 ppm, it had a mortality of 90% of the 2nd-instar larvae. It decreased pupal weight by inhibition digestion or post absorption. Higher activity of detoxifying enzymes and increase in oxidative stress markers of larvae when compared to control.	[63]
Genistein	*A. pisum*	Pea and other leguminous	Prolonged period of probing and shortened passive digestion duration. Passive ingestion and salivation were completely blocked at 1000 µg/cm^3^.	[45]
			At 1 and 10 µg/cm^3^, it reduced the survival rate of 2nd-instar nymphs of *Pisum* host race after 5 days. It did not affect the 2nd-instar nymphs of *Medicago* host race.	[43]
			At 0.1%, it did not significantly affect aphid probing behavior.	[56]
	*Oedaleus asiaticus* (Bey-Bienko, 1941)	Grassland forages	At 5 μg/μL, it reduced the survival rate after 8 days, weight, and growth of 5th-instar nymphs of *Oedaleus asiaticus* when compared with control and PTP1B-IN-1. It negatively regulates insulin-signaling pathway by inhibiting protein tyrosine kinase, resulting in suppressed growth and development.	[49]

## Data Availability

No new data were created or analyzed in this study. Data sharing is not applicable to this article.

## References

[1] García-Lara S., Saldivar S.O.S., Caballero B., Finglas P.M., Toldrá F. (2016). Insect Pests. Encyclopedia of Food and Health.

[2] Manosathiyadevan M., Bhuvaneshwari V., Latha R., Dhanarajan A. (2017). Impact of Insects and Pests in Loss of Crop Production: A Review. Sustainable Agriculture towards Food Security.

[3] Struik P.C., Kuyper T.W. (2017). Sustainable Intensification in Agriculture: The Richer Shade of Green. A Review. Agron. Sustain. Dev..

[4] Narayan S., Liew Z., Bronstein J.M., Ritz B. (2017). Occupational Pesticide Use and Parkinson’s Disease in the Parkinson Environment Gene (PEG) Study. Environ. Int..

[5] Torres-Sánchez E.D., Ortiz G.G., Reyes-Uribe E., Torres-Jasso J.H., Salazar-Flores J. (2023). Effect of Pesticides on Phosphorylation of Tau Protein, and Its Influence on Alzheimer’s Disease. World J. Clin. Cases.

[6] Kass L., Gomez A.L., Altamirano G.A. (2020). Relationship between Agrochemical Compounds and Mammary Gland Development and Breast Cancer. Mol. Cell. Endocrinol..

[7] Khan U.M., Sameen A., Aadil R.M., Shahid M., Sezen S., Zarrabi A., Ozdemir B., Sevindik M., Kaplan D.N., Selamoglu Z. (2021). Citrus Genus and Its Waste Utilization: A Review on Health-Promoting Activities and Industrial Application. Evid-Based Complement. Altern. Med..

[8] Kim B., Park E.Y., Kim J., Park E., Oh J.-K., Lim M.K. (2022). Occupational Exposure to Pesticides and Lung Cancer Risk: A Propensity Score Analyses. Cancer Res. Treat..

[9] Matich E.K., Laryea J.A., Seely K.A., Stahr S., Su L.J., Hsu P.-C. (2021). Association between Pesticide Exposure and Colorectal Cancer Risk and Incidence: A Systematic Review. Ecotoxicol. Environ. Saf..

[10] Pardo L.A., Beane Freeman L.E., Lerro C.C., Andreotti G., Hofmann J.N., Parks C.G., Sandler D.P., Lubin J.H., Blair A., Koutros S. (2020). Pesticide Exposure and Risk of Aggressive Prostate Cancer among Private Pesticide Applicators. Environ. Health.

[11] Varghese J.V., Sebastian E.M., Iqbal T., Tom A.A. (2021). Pesticide Applicators and Cancer: A Systematic Review. Rev. Environ. Health.

[12] Fucic A., Duca R.C., Galea K.S., Maric T., Garcia K., Bloom M.S., Andersen H.R., Vena J.E. (2021). Reproductive Health Risks Associated with Occupational and Environmental Exposure to Pesticides. Int. J. Environ. Res. Public Health.

[13] Tudi M., Li H., Li H., Wang L., Lyu J., Yang L., Tong S., Yu Q.J., Ruan H.D., Atabila A. (2022). Exposure Routes and Health Risks Associated with Pesticide Application. Toxics.

[14] Venkidasamy B., Subramanian U., Samynathan R., Rajakumar G., Shariati M.A., Chung I.-M., Thiruvengadam M. (2021). Organopesticides and Fertility: Where Does the Link Lead To?. Environ. Sci. Pollut. Res..

[15] Campos E.V.R., Proença P.L.F., Oliveira J.L., Bakshi M., Abhilash P.C., Fraceto L.F. (2019). Use of Botanical Insecticides for Sustainable Agriculture: Future Perspectives. Ecol. Indic..

[16] Rasool S., Rasool T., Gani K.M. (2022). A Review of Interactions of Pesticides within Various Interfaces of Intrinsic and Organic Residue Amended Soil Environment. Chem. Eng. J. Adv..

[17] Syafrudin M., Kristanti R.A., Yuniarto A., Hadibarata T., Rhee J., Al-Onazi W.A., Algarni T.S., Almarri A.H., Al-Mohaimeed A.M. (2021). Pesticides in Drinking Water—A Review. Int. J. Environ. Res. Public Health.

[18] Gui F., Lan T., Zhao Y., Guo W., Dong Y., Fang D., Liu H., Li H., Wang H., Hao R. (2022). Genomic and Transcriptomic Analysis Unveils Population Evolution and Development of Pesticide Resistance in Fall Armyworm *Spodoptera frugiperda*. Protein Cell.

[19] Zhang Y., Xu D., Zhang Y., Wu Q., Xie W., Guo Z., Wang S. (2022). Frequencies and Mechanisms of Pesticide Resistance in *Tetranychus urticae* Field Populations in China. Insect Sci..

[20] Li B.A., Li B.M., Bao Z., Li Q., Xing M., Li B. (2023). Dichlorodiphenyltrichloroethane for Malaria and Agricultural Uses and Its Impacts on Human Health. Bull. Environ. Contam. Toxicol..

[21] Nollet L.M.L., Rathore H.S. (2015). Biopesticides Handbook.

[22] Tataridas A., Kanatas P., Chatzigeorgiou A., Zannopoulos S., Travlos I. (2022). Sustainable Crop and Weed Management in the Era of the EU Green Deal: A Survival Guide. Agronomy.

[23] Tavares W.R., Barreto M.D.C., Seca A.M.L. (2021). Aqueous and Ethanolic Plant Extracts as Bio-Insecticides—Establishing a Bridge between Raw Scientific Data and Practical Reality. Plants.

[24] Karak P. (2019). Biological Activities of Flavonoids: An Overview. Int. J. Pharm. Sci. Res..

[25] Mathesius U. (2018). Flavonoid Functions in Plants and Their Interactions with Other Organisms. Plants.

[26] Baskar V., Venkatesh R., Ramalingman S., Gulpta D., Palma J., Corpas F. (2018). Flavonoids (Antioxidants Systems) in Higher Plants and Their Response to Stresses. Antioxidants and Antioxidant Enzymes in Higher Plants.

[27] Liu W., Feng Y., Yu S., Fan Z., Li X., Li J., Yin H. (2021). The Flavonoid Biosynthesis Network in Plants. Int. J. Mol. Sci..

[28] Yonekura-Sakakibara K., Higashi Y., Nakabayashi R. (2019). The Origin and Evolution of Plant Flavonoid Metabolism. Front. Plant Sci..

[29] Schnarr L., Segatto M.L., Olsson O., Zuin V.G., Kümmerer K. (2022). Flavonoids as Biopesticides—Systematic Assessment of Sources, Structures, Activities and Environmental Fate. Sci. Total Environ..

[30] Wang J., Li G., Li C., Zhang C., Cui L., Ai G., Wang X., Zheng F., Zhang D., Larkin R.M. (2021). NF-Y Plays Essential Roles in Flavonoid Biosynthesis by Modulating Histone Modifications in Tomato. New Phytol..

[31] Yin Y., Zhang X., Gao Z., Hu T., Liu Y. (2019). The Research Progress of Chalcone Isomerase (CHI) in Plants. Mol. Biotechnol..

[32] Grotewold E. (2006). The Genetics and Biochemistry of Floral Pigments. Annu. Rev. Plant Biol..

[33] Veremeichik G.N., Grigorchuk V.P., Butovets E.S., Lukyanchuk L.M., Brodovskaya E.V., Bulgakov D.V., Bulgakov V.P. (2021). Isoflavonoid Biosynthesis in Cultivated and Wild Soybeans Grown in the Field under Adverse Climate Conditions. Food Chem..

[34] Wang L., Lui A.C.W., Lam P.Y., Liu G., Godwin I.D., Lo C. (2020). Transgenic Expression of Flavanone 3-Hydroxylase Redirects Flavonoid Biosynthesis and Alleviates Anthracnose Susceptibility in Sorghum. Plant Biotechnol. J..

[35] Zuk M., Szperlik J., Hnitecka A., Szopa J. (2019). Temporal Biosynthesis of Flavone Constituents in Flax Growth Stages. Plant Physiol. Biochem..

[36] Meng X., Li Y., Zhou T., Sun W., Shan X., Gao X., Wang L. (2019). Functional Differentiation of Duplicated Flavonoid 3-O-Glycosyltransferases in the Flavonol and Anthocyanin Biosynthesis of *Freesia hybrida*. Front. Plant Sci..

[37] Yan H., Pei X., Zhang H., Li X., Zhang X., Zhao M., Chiang V.L., Sederoff R.R., Zhao X. (2021). Myb-Mediated Regulation of Anthocyanin Biosynthesis. Int. J. Mol. Sci..

[38] Brugliera F., Tao G.Q., Tems U., Kalc G., Mouradova E., Price K., Stevenson K., Nakamura N., Stacey I., Katsumoto Y. (2013). Violet/Blue Chrysanthemums-Metabolic Engineering of the Anthocyanin Biosynthetic Pathway Results in Novel Petal Colors. Plant Cell Physiol..

[39] Lepiniec L., Debeaujon I., Routaboul J.M., Baudry A., Pourcel L., Nesi N., Caboche M. (2006). Genetics and Biochemistry of Seed Flavonoids. Annu. Rev. Plant Biol..

[40] Zhu H., Yang J., Xiao C., Mao T., Zhang J., Zhang H. (2019). Differences in Flavonoid Pathway Metabolites and Transcripts Affect Yellow Petal Colouration in the Aquatic Plant *Nelumbo nucifera*. BMC Plant Biol..

[41] Li W., Ding Y., Qi H., Liu T., Yang Q. (2021). Discovery of Natural Products as Multitarget Inhibitors of Insect Chitinolytic Enzymes through High-Throughput Screening. J. Agric. Food Chem..

[42] Puri S., Singh S., Sohal S.K. (2022). Inhibitory Effect of Chrysin on Growth, Development and Oviposition Behaviour of Melon Fruit Fly, *Zeugodacus cucurbitae* (Coquillett) (Diptera: Tephritidae). Phytoparasitica.

[43] Yuan E., Yan H., Gao J., Guo H., Ge F., Sun Y. (2019). Increases in Genistein in *Medicago sativa* Confer Resistance against the *Pisum* Host Race of *Acyrthosiphon pisum*. Insects.

[44] Goławska S., Sprawka I., Łukasik I., Goławski A. (2014). Are Naringenin and Quercetin Useful Chemicals in Pest-Management Strategies?. J. Pest Sci..

[45] Goławska S., Łukasik I. (2012). Antifeedant Activity of Luteolin and Genistein against the Pea Aphid, *Acyrthosiphon pisum*. J. Pest Sci..

[46] Sarria F.A.L., Matos A.P., Volante A.C., Bernardo A.R., Sabbag Cunha G.O., Fernandes J.B., Rossi Forim M., Vieira P.C., da Silva M.F.D.G.F. (2022). Insecticidal Activity of Copper (II) Complexes with Flavanone Derivatives. Nat. Prod. Res..

[47] Qi S.H., Zhang S., Qian P.Y., Wang B.G. (2008). Antifeedant, Antibacterial, and Antilarval Compounds from the South China Sea Seagrass *Enhalus acoroides*. Bot. Mar..

[48] Zhao C., Ma C., Luo J., Niu L., Hua H., Zhang S., Cui J. (2021). Potential of Cucurbitacin B and Epigallocatechin Gallate as Biopesticides against *Aphis gossypii*. Insects.

[49] Chang B.H., Qiang B., Li S., Ullah H., Hao K., McNeill M.R., Rajput A., Raza A., Huang X., Zhang Z. (2020). Inhibitory Effect of Genistein and PTP1B on Grasshopper *Oedaleus asiaticus* Development. Arthropod. Plant. Interact..

[50] Wiwattanawanichakun P., Saehlee S., Yooboon T., Kumrungsee N., Nobsathian S., Bullangpoti V. (2022). Toxicity of Isolated Phenolic Compounds from *Acorus calamus* L. to Control *Spodoptera litura* (Lepidoptera: Noctuidae) under Laboratory Conditions. Chem. Biol. Technol. Agric..

[51] Zhang X.-Y., Shen J., Zhou Y., Wei Z.-P., Gao J.-M. (2017). Insecticidal Constituents from *Buddlej aalbiflora* Hemsl. Nat. Prod. Res..

[52] Ruttanaphan T., Songoen W., Pluempanupat W., Bullangpoti V. (2023). Potential Insecticidal Extracts from *Artocarpus lacucha* against *Spodoptera litura* (Lepidoptera: Noctuidae) Larvae. J. Econ. Entomol..

[53] Da Silva D., Bomfim J., Marchi R., Amaral J., Pinto L., Carlos R., Ferreira A., Forim M., Fernandes J., da Silva M. (2022). Valorization of Hesperidin from Citrus Residues: Evaluation of Microwave-Assisted Synthesis of Hesperidin-Mg Complex and Their Insecticidal Activity. J. Braz. Chem. Soc..

[54] Diaz Napal G.N., Defagó M.T., Valladares G.R., Palacios S.M. (2010). Response of Epilachna Paenulata to Two Flavonoids, Pinocembrin and Quercetin, in a Comparative Study. J. Chem. Ecol..

[55] Diaz Napal G.N., Palacios S.M. (2015). Bioinsecticidal Effect of the Flavonoids Pinocembrin and Quercetin against Spodoptera Frugiperda. J. Pest Sci..

[56] Stec K., Kordan B., Gabryś B. (2021). Effect of Soy Leaf Flavonoids on Pea Aphid Probing Behavior. Insects.

[57] Datta R., Kaur A., Saraf I., Singh I.P., Kaur S. (2019). Effect of Crude Extracts and Purified Compounds of *Alpinia galanga* on Nutritional Physiology of a Polyphagous Lepidopteran Pest, *Spodoptera litura* (Fabricius). Ecotoxicol. Environ. Saf..

[58] Stec K., Kordan B., Gabryś B. (2021). Quercetin and Rutin as Modifiers of Aphid Probing Behavior. Molecules.

[59] Sharma R., Sohal S.K. (2013). Bioefficacy of Quercetin against Melon Fruit Fly. Bull. Insectology.

[60] Jadhav D.R., Mallikarjuna N., Rathore A., Pokle D. (2012). Effect of Some Flavonoids on Survival and Development *of Helicoverpa armigera* (Hübner) and *Spodoptera litura* (Fab) (Lepidoptera: Noctuidae). Asian J. Agric. Sci..

[61] Selin-Rani S., Senthil-Nathan S., Thanigaivel A., Vasantha-Srinivasan P., Edwin E.-S., Ponsankar A., Lija-Escaline J., Kalaivani K., Abdel-Megeed A., Hunter W.B. (2016). Toxicity and Physiological Effect of Quercetin on Generalist Herbivore, *Spodoptera litura* Fab. and a Non-Target Earthworm Eisenia Fetida Savigny. Chemosphere.

[62] Zhou Y.Y., Luo S.H., Yi T.S., Li C.H., Luo Q., Hua J., Liu Y., Li S.H. (2011). Secondary Metabolites from Glycine Soja and Their Growth Inhibitory Effect against *Spodoptera litura*. J. Agric. Food Chem..

[63] Punia A., Chauhan N.S. (2022). Effect of Daidzein on Growth, Development and Biochemical Physiology of Insect Pest, *Spodoptera litura* (Fabricius). Comp. Biochem. Physiol. Part C Toxicol. Pharmacol..

[64] Ohmura W., Doi S., Aoyama M., Ohara S. (2000). Antifeedant Activity of Flavonoids and Related Compounds against the Subterranean Termite *Coptotermes formosanus* Shiraki. J. Wood Sci..

[65] Morimoto M., Tanimoto K., Nakano S., Ozaki T., Nakano A., Komai K. (2003). Insect Antifeedant Activity of Flavones and Chromones against *Spodoptera litura*. J. Agric. Food Chem..

[66] War A.R., Paulraj M.G., Hussain B., Buhroo A.A., Ignacimuthu S., Sharma H.C. (2013). Effect of Plant Secondary Metabolites on Legume Pod Borer, *Helicoverpa armigera*. J. Pest Sci..

[67] Su Q., Zhou Z., Zhang J., Shi C., Zhang G., Jin Z., Wang W., Li C. (2018). Effect of Plant Secondary Metabolites on Common Cutworm, *Spodoptera litura* (Lepidoptera: Noctuidae). Entomol. Res..

[68] Mikani A. (2019). Effect of Quercetin on Some Digestive Enzyme Activity via Crustacean Cardioactive Peptide (CCAP) Content of the Midgut of the Diamondback Moth, *Plutella xylostella* (Lepidoptera: Plutellidae). J. Crop Prot..

[69] Maazoun A.M., Hamdi S.H., Belhadj F., Jemâa J.M.B., Messaoud C., Marzouki M.N. (2019). Phytochemical Profile and Insecticidal Activity of *Agave americana* Leaf Extract towards *Sitophilus oryzae* (L.) (Coleoptera: Curculionidae). Environ. Sci. Pollut. Res..

[70] Wang Z., Zhao Z., Cheng X., Liu S., Wei Q., Scott I.M. (2016). Conifer Flavonoid Compounds Inhibit Detoxification Enzymes and Synergize Insecticides. Pestic. Biochem. Physiol..

[71] Li M., Gao X., Lan M., Liao X., Su F., Fan L., Zhao Y., Hao X., Wu G., Ding X. (2020). Inhibitory Activities of Flavonoids from Eupatorium Adenophorum against Acetylcholinesterase. Pestic. Biochem. Physiol..

[72] Kumar M., Gupta G.P., Rajam M.V. (2009). Silencing of Acetylcholinesterase Gene of *Helicoverpa armigera* by SiRNA Affects Larval Growth and Its Life Cycle. J. Insect Physiol..

[73] Haribal M., Renwick J.A.A. (1996). Oviposition Stimulants for the Monarch Butterfly: Flavonol Glycosides from *Asclepias curassavica*. Phytochemistry.

[74] Honda K. (1986). Flavanone Glycosides as Oviposition Stimulants in a Papilionid Butterfly, Papilio Protenor. J. Chem. Ecol..

[75] Riddick E., Wu Z., Eller F., Berhow M. (2018). Utilization of Quercetin as an Oviposition Stimulant by Lab-Cultured *Coleomegilla maculata* in the Presence of Conspecifics and a Tissue Substrate. Insects.

[76] Isaac R.E., Ekbote U., Coates D., Shirras A.D. (2006). Insect Angiotensin-converting Enzyme: A Processing Enzyme with Broad Substrate Specificity and a Role in Reproduction. Ann. N. Y. Acad. Sci..

[77] Shinoda T., Itoyama K. (2003). Juvenile Hormone Acid Methyltransferase: A Key Regulatory Enzyme for Insect Metamorphosis. Proc. Natl. Acad. Sci. USA.

[78] Chen W., Yang Q. (2020). Development of Novel Pesticides Targeting Insect Chitinases: A Minireview and Perspective. J. Agric. Food Chem..

[79] Hussain A., AlJabr A.M., Al-Ayedh H. (2019). Development-Disrupting Chitin Synthesis Inhibitor, Novaluron, Reprogramming the Chitin Degradation Mechanism of Red Palm Weevils. Molecules.

[80] Ren Y., Li Q., Lu L., Jin H., Tao K., Hou T. (2021). Toxicity and Physiological Actions of Biflavones on Potassium Current in Insect Neuronal Cells. Pestic. Biochem. Physiol..

[81] Andersch W., Hungenberg H., Mansfield D. (2009). Insecticidal Active Ingredient Combinations (Formononetins + Insecticides).

[82] Pang Y.P., Cohen E. (2014). Chapter Six—Insect Acetylcholinesterase as a Target for Effective and Environmentally Safe Insecticides. Advances in Insect Physiology.

[83] Ozan A., Safir G.R., Nair M.G. (1997). Persistence of Isoflavones Formononetin and Biochanin A in Soil and Their Effects on Soil Microbe Populations. J. Chem. Ecol..

[84] Shaw L.J., Hooker J.E. (2008). The Fate and Toxicity of the Flavonoids Naringenin and Formononetin in Soil. Soil Biol. Biochem..

[85] Nuruzzaman M., Rahman M.M., Liu Y., Naidu R. (2016). Nanoencapsulation, Nano-Guard for Pesticides: A New Window for Safe Application. J. Agric. Food Chem..

